# Characterization of Room-Temperature Ionic Liquids to Study the Electrochemical Activity of Nitro Compounds

**DOI:** 10.3390/s20041124

**Published:** 2020-02-19

**Authors:** Ivneet Banga, Anirban Paul, Sriram Muthukumar, Shalini Prasad

**Affiliations:** 1 Department of Bioengineering, University of Texas at Dallas, Richardson, TX 75080, USA; ivneetkaur.banga@utdallas.edu (I.B.); anirban.paul@utdallas.edu (A.P.); sriramm@enlisense.com (S.M.); 2 EnLiSense LLC, 1813 Audubon Pondway, Allen, TX 75013, USA

**Keywords:** room temperature ionic liquids, square wave voltammetry, electrochemical sensor, nitroaromatics

## Abstract

Over the past few years, room-temperature ionic liquid (RTIL) has evolved as an important solvent-cum-electrolyte because of its high thermal stability and excellent electrochemical activity. Due to these unique properties, RTILs have been used as a solvent/electrolyte/mediator in many applications. There are many RTILs, which possess good conductivity as well as an optimal electrochemical window, thus enabling their application as a transducer for electrochemical sensors. Nitroaromatics are a class of organic compounds with significant industrial applications; however, due to their excess use, detection is a major concern. The electrochemical performance of a glassy carbon electrode modified with three different RTILs, [EMIM][BF_4_], [BMIM][BF_4_] and [EMIM][TF_2_N], has been evaluated for the sensing of two different nitroaromatic analytes: 2,6-dinitrotoluene (2,6 DNT) and ethylnitrobenzene (ENB). Three RTILs have been chosen such that they have either a common anion or cation amongst them. The sensory response has been measured using square wave voltammetry (SQWV). We found the transducing ability of [EMIM][BF_4_] to be superior compared to the other two RTILs. A low limit of detection (LOD) of 1 ppm has been achieved with a 95% confidence interval for both the analytes. The efficacy of varying the cationic and anionic species of RTIL to obtain a perfect combination has been thoroughly investigated in this work, which shows a novel selection process of RTILs for specific applications. Moreover, the results obtained from testing with a glassy carbon electrode (GCE) have been replicated using a miniaturized sensor platform that can be deployed easily for on-site sensing applications.

## 1. Introduction

Room-temperature ionic liquids (RTILs) are an exciting class of solvents that have advanced as sensing modalities [1]. Ionic liquids are unique compounds, originally classified, based on their boiling point with respect to water, in that they are liquid at room temperatures below 100 °C [2]. Room-temperature ionic liquids consist of a bulky cation and an organic/inorganic anion comprising selective molten salts or oxides. The compound was first used in 1914 when Walden synthesized a low melting-point salt of ethyl ammonium nitrate (melting point 12 °C) [3]. The chemical structure of ionic liquids allows many non-covalent interactions to stabilize important molecules which are difficult to keep stable in conventional solvents. “Room-temperature ionic liquid” was coined much later by Earl and Seddon [4]. Until then, more than 500 different types of RTIL had been synthesized, yielding thousands of permutations and combinations of different anions and cations [5]. The most interesting property of the RTIL is its electro neutrality, consisting of two exact opposite charged species held together with strong ionic interactions. Physically, it has very low vapor pressure and density higher than water. Due to their negligible vapor pressure, the thermal stability of RTILs is reported to be very high (up to 400 K), enabling them to be used in a variety of applications such as sensing, catalysis, high temperature conversion processes, and process development among others. Our group has recently published a review article discussing the fundamental principles and applications of RTILs in electrochemical sensing [6]. 

RTIL is generally a combination of a large asymmetric organic cation and a small organic/inorganic anion present in a molten state. There are many reported synthetic RTILs, among which, functionalized imidazolium, pyridinium, quaternary ammonium salts can be found as popular cation choices whereas halides, tetrafluoroborate(BF_4_^−^), tetrachloroaluminate (AlCl_4_^−^), hexafluorophosphate (PF_6_^−^), bis (perfluoromethylsulphonyl) imide/bistriflate imide (TF_2_N) and trifluoromethanesulphonate (F_3_MeSO_4_^−^) are used as common anions of RTIL [7,8,9,10,11,12,13]. There is a wide array of compounds that can be synthesized to leverage the physicochemical properties of RTILs through proper selection of cationic and anionic groups. Regardless of such potential, there is still an inadequate number of RTILs available for specific trouble shooting in electrochemical regimes.

Most of the reportedly synthesized RTILs possess low conductivity (<5 mS/cm) and high viscosity (>50 cP). This inhibits their applicability in electrochemical sensing, as the conductivity is one of the driving parameters in electrochemistry. To fabricate a good electrochemical sensor, one should expect RTIL to possess good conductivity (>5 mS/cm) and to have a good electrochemical stability window of ±2.5 V [8]. An important hypothesis introduced by Walden suggests that a long alkyl-chain hydrocarbon cation-based RTIL possesses reduced conductivity [14]. Based on this hypothesis, experimental reports have shown a trend of ionic conductivity of both cations and anions, which are as follows:

Cations:


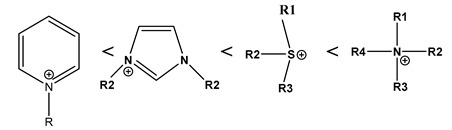


Whereas the anions follow this order:

$${\mathrm{Hal}}^{-}<{\mathrm{AlCl}}_{4}^{-}<{\mathrm{PF}}_{6}^{-},{\mathrm{BF}}_{4}^{-}<N^{-}{\left({\mathrm{SO}}_{2}{\mathrm{CF}}_{3}\right)}_{2}$$

Several combinations are possible by selecting different cationic and anionic groups such that the synthesized RTIL can be used for specific detection methods and as a transducing element in a chemical sensor. RTIL is liquid in nature and a binder is necessary to hold the species over the electrode surface. Nafion is a common ion-exchange polymer, widely used to bind species over the surface of the electrodes [15,16]. It is semi-permeable in nature and it is well known for its excellent proton conductivity. Doping of the ionic liquid with a fixed amount of Nafion can help improve the ionic conductivity as well as allowing for better adhesion onto the surface of the glassy carbon electrode (GCE) [17]. Chen et al. [18] reported the use of nafion to bind1-Butyl-3-methylimidazolium hexafluorophosphate [BMIM][PF_6_] onto the surface of a glassy carbon electrode and performed electrochemical characterization of the composite thin film so formed. The results obtained clearly showed that the thin film adhered onto the GCE surface. Moreover, it was found that doping of the ionic liquid in nafion reduces the electron transfer resistance and, thus, nafion can be employed as an immobilization matrix to entrap the oxidized or reduced species [18]. For this purpose, it is widely used to bind mesoporous materials or nanomaterials [19,20,21,22,23,24,25,26,27]. 

In this report, our goal is to evaluate the electrochemical activity of specific RTILs to develop an electrochemical transduction platform for the evaluation of nitroaromatic compounds. We selected three different RTILs with either a common cation or anion and used a few important nitroaromatic compounds to provide the proof of concept for the signal transduction characterization of RTIL toward these compounds.

Nitroaromatics, or compounds containing a nitro group attached to benzene or functionalized benzene groups, are well known for their toxic effects as pollutants but are extensively used in explosives, fertilizers, and many industrial processes [28]. There is a significant effort to detect the presence of these compounds in low concentration. Colorimetric estimation of nitro explosives is one of the most commonly used method for on-field detection where the concentration of these nitroaromatics is either directly or indirectly measured [29,30,31,32,33,34,35,36,37,38,39,40,41,42,43,44,45]. Although there is a lot of advancement in this field, these technologies are limited due to the requirement of heavy and sophisticated instrumentation, which prohibits them from being candidates for the development of a low-cost field applicable Internet of Things (IoT)-based devices. Electrochemical detection mechanisms, on the other hand, can be deigned to be field-applicable, robust, small form-factor, IoT compatible, environmentally friendly, low power, lower resources, and simple to use device. The use of RTILs in electrochemical sensing is not new and we have reviewed the existing research in the field; however, not much was available on the use of RTILs as a mediator/transducer for fabrication of electrochemical chemo sensor of important nitro compounds. 

We have varied both the cation and anion of RTIL species and found the effects on the electrode-electrolyte interface in a specific electrochemical signal transduction scenario. We have used three different RTILs: 1-butyl-3-methyl imidazolium tetrafluoroborate [BMIM][BF_4_], 1-ethyl-3-methyl imidazolium tetrafluoroborate [EMIM][BF_4_] and 1-ethyl-3-methyl imidazolium bis-(trifluoromethylsulphonyl) imide [EMIM][TF_2_N] coated on a standard glassy carbon electrode and found concentration-dependent sensor responses for two important nitroaromatics: 2,6-dinitroluene and ethyl nitrobenzene. Square wave voltammetry (SQWV) has been performed to obtain specific current changes of these compounds as we are looking for the electrochemical reduction of the –NO_2_ group. We found that among these three RTILs, [EMIM][BF_4_] showed the most promising results and can be used as transuding element for signal amplification as compared to the other two. The superiority of this species was predicted due to its specific physico-chemical properties, which can be attributed to the presence of cationic and anionic species. A low detection of 1 ppm was found for both species. This is the first report where the two ionic species (cationic and anionic) s have been varied to obtain an optimal electrochemical signal transduction, which is effective for the detection of nitroaromatics.

## 2. Materials and Methods

Ionic liquids such as 1-ethyl-3-methylimidazolium tetrafluoroborate [EMIM][BF_4_], 1-butyl-3-methylimidazolium tetrafluoroborate [BMIM][BF_4_], and 1-ethyl-3-methylimidazolium bis-(trifluoromethylsulfonyl) imide [EMIM][TF_2_N] with over 97% purity and for electrochemistry purposes were obtained from Milipore Sigma. The crosslinking polymer such as nafion, as a 5% wt solution in a mixture of lower aliphatic alcohols and 10% water, was purchased from Aldrich. Unless otherwise stated, 0.1 M potassium chloride (KCl) was used as supporting electrolyte in all studies. 

A cross-linked network of nafion and RTIL was prepared by mixing equal volumes of RTIL and nafion solution together, and further dissolving the mixture into methanol. The solution was sonicated for 10 min leading to the formation of a cross-linked network of nafion-RTIL, which was further drop-casted onto the electrode surface and allowed to dry at room temperature. This resulted in the formation of a thin-film structure onto the electrode surface that allowed for diffusion of the target analytes to take place; 0.1 M KCl was used as a supporting electrolyte to carry out the bare electrode experiments, that is, an electrode without a ionic liquid-nafion network. Analytes such as 2,6-dinitrotoluene (2,6 DNT) and ethylnitrobenzene (ENB) were purchased from Milipore Sigma and were used as is. The stock solution (2000 ppm) for the analytes were prepared in methanol and the dilutions (1 ppm–1000 ppm) were prepared using 0.1 M KCl solution. Safety considerations were followed when dealing with nitroaromatic compounds since they are highly toxic in nature. All the samples were prepared and handled inside the fume-hood Proper protective equipment (PPE) was worn to avoid skin and eye contact and to prevent accidental inhalation and digestion. The experiments were repeated at least three times and a fresh GCE was used for each analyte of interest.

### 2.1. Electrochemical Methods

A standard three-electrode configuration was used for electrochemical experiments. A GCE of 3 mm diameter was used as the working electrode (WE) Platinum wire was used as the counter electrode (CE) whereas Ag/AgCl (sat. KCl) was used as the reference electrode (RE). The electrochemical cell setup was purchased from CH instruments. The electrochemical experiments were performed using the Gamry series 600 potentiostat/galvanostat. Cyclic voltammetry (CV) was performed in a range of −1.2 V to +1.2 V vs. Ag/AgCl (sat. KCl) with a scan rate of 50 mV/S. Scan rate was varied from 50 mV/S to 250 mV/S to obtain the specific capacitance of the RTIL-modified GCE interface. Square wave voltammetry (SQWV) was performed to obtain calibrated dose response. SQWV was performed in a range of +0.4 V to −1.2 V vs. Ag/AgCl (sat. KCl) with an amplitude of 25 mV, step size of 5 mV and frequency is 25 Hz. Double potential chrono-amperometry (CA) was performed at a fixed potential of −0.8 V for 30 s and +0.8 V for 30 s. Prior to each experiment, the GCE was polished using 0.4-micron alumina mesh and washed thoroughly with DI water and methanol. The electrode was then dried under the flow of nitrogen to remove any unwanted residues. The platinum counter electrode was washed using the same method. The reference electrode was only washed with deionized (DI) water and kept in saturated KCl solution to avoid any chemical contamination.

### 2.2. Electrode Preparation

The GCE was placed in an upward orientation and 10 microliters of RTIL (100%) mixed with nafion, was dissolved in methanol, and directly drop casted at the surface of the electrode. The electrode was kept for 15 min at room temperature to dry. A thin film of the desired RTIL-nafion was observed at the electrode surface. The surface-modified GCE was then directly immersed into different concentrations of analyte solutions to obtain CV, SQWV, and CA. The electrode was washed thoroughly after finishing each experiment.

The experimental platform was miniaturized using a set of interdigitated electrodes (IDE) to allow for incorporation into a handheld device. The surface of the IDE was modified with the RTIL-nafion network by drop casting 3 mL of RTIL (100%) mixed with Nafion and dissolved in methanol. This was allowed to dry at room temperature so that a thin-film network was formed. The surface-modified IDE was used to detect low, medium, and high doses of nitroaromatic compounds to study the trend in detection current. Double potential chronoamperometry was performed at −0.8 V for 30 s and −0.8 V for 30 s. Target analyte concentrations of 1, 500, and 1000 ppm were chosen for both ENB and 2,6 DNT. 

## 3. Results and Discussion

### 3.1. Selection of Room-Temperature Ionic Liquid (RTIL)

**Figure 1:**
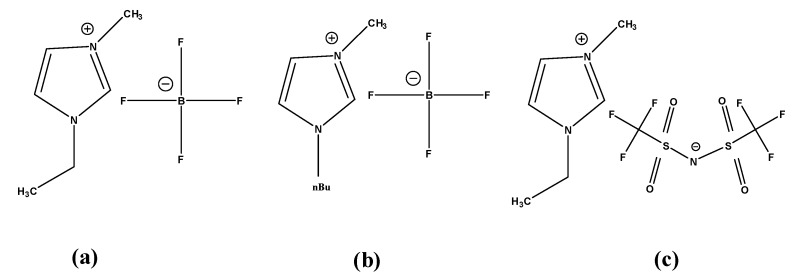
(**a**) 1-Ethyl-3-methylimidazolium tetrafluoroborate [EMIM][BF_4_], (**b**) 1-butyl-3-methylimidazolium tetrafluoroborate [BMIM][BF_4_] and (**c**) 1-ethyl-3-methylimidazolium bis (trifluoromethylsulfonyl) imide [EMIM][TF_2_N].

Based on the hypothesis suggested by Walden, many RTILs have been synthesized. Among them, cations like BMIM^+^, EMIM^+^ and anions like BF_4_^−^ and TF_2_N^−^ are highly popular for their high conductivity, electrochemical stability, and moderate viscosity. To compare the physico-chemical properties as a function of cation and anion species, we have prepared three different RTILs with varying combinations of one cation and one anion. The structure of these three RTILs has been depicted in Figure 1.

The physical properties of these three RTILs are described in Table 1.

Table 1 reflects the superiority of [EMIM][BF_4_] over the other two ionic liquids. Nonetheless, we cannot infer from this table which species has better transducing abilities in an electrode–electrolyte interface. To obtain a deeper perspective of the preferable attributes in each RTIL, we have gone through a conventional electrochemical characterization process as described stepwise.

### 3.2. Cyclic Voltammetry (CV) Characterization of Different RTILs

**Figure 2:**
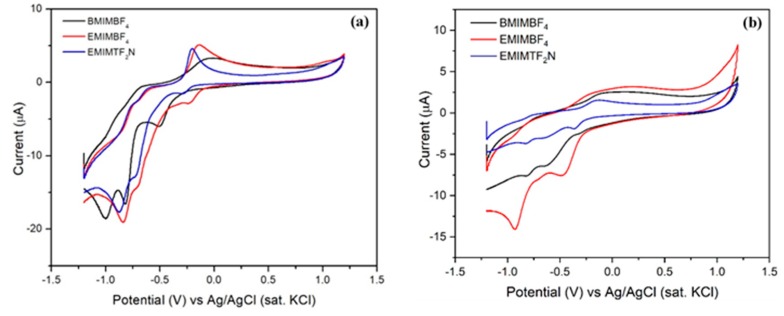
Cyclic voltammetry (CV) was undertaken with RTIL-modified glassy carbon electrode (GCE) as WE, Pt wire as CE and Ag/AgCl (sat. KCl) as RE, using two different analytes: 2,6-dinitrotoluene (2,6 DNT) and ethylnitrobenzene (ENB). Scan rate 50 mV/S. Cyclic voltammogram of (**a**) 2, 6 DNT and (**b**) ENB showing distinct peaks for nitro reduction.

CV is an important technique that is often used for the characterization of the electrode electrolyte reaction. Due to the high ionic conductivity of RTIL, the hybrid interface of RTIL@GCE is an extremely important aspect to be evaluated. For this purpose, CV was performed with the three different variations of RTIL coated onto GCE in a range of −1.2 V to +1.2 V vs. Ag/AgCl (sat. KCl). The range was confined within this regime to avoid electrolysis of water after 1.23 V. The scan rate was chosen to be 50 mV/S. The concentration of the target analyte was 100 ppm. 2,6 DNT and ENB are dissolved in 0.1 M KCl and CV was performed. The cyclic voltammogram for 2,6 DNT and ENB is depicted in Figure 2.

The results show multiple peaks for 2,6 DNT and ENB. The peaks are depicted in Table 2 and Table 3 for 2,6 DNT and ENB respectively. 

**Figure 3:**
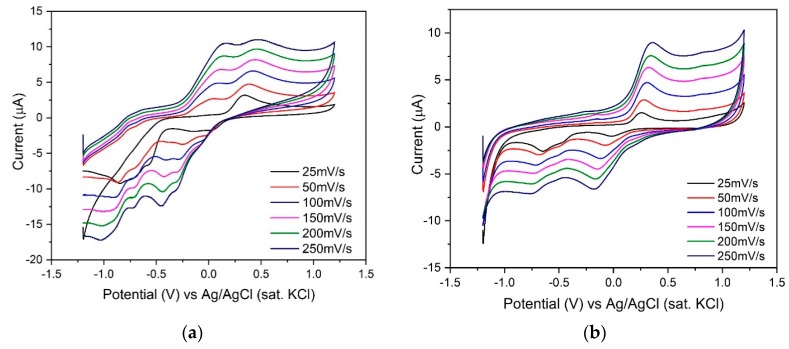
Scan rate has been varied from 25 mV/S to 250 mV/S using [EMIM][BF_4_]-nafion @GCE in two different analytes. (**a**) CV with different scan rate along with the change of peak current with scan rate (inset) of 2,6 DNT; (**b**) CV with different scan rate along with the change of peak current with scan rate (inset) of ENB.

Based on the results obtained from cyclic voltammetry, [EMIM][BF_4_] possesses sharp cathodic as well as anodic peak current response compared to its isomorphs. Although the CV response of other cations like [BMIM] and anions like [TF_2_N] are quite similar to [EMIM][BF_4_], the higher peak current intensity of [EMIM][BF_4_] is definitely due to its optimal electronic architecture that results in higher ionic conductivity, ultimately reflected in the CV output [46]. Moreover, the sensor surface was characterized in the absence of the target species to understand the electrochemical activity of RTIL only using 0.1 M KCl solution. The data obtained are depicted in Figure S1 in the Supplementary Materials. They show an almost similar area under the curve for [BMIM][BF_4_] and [EMIM][BF_4_] but [EMIM][TF_2_N] possesses a very small area under the curve. The result obtained further substantiates our previous experiment with target analytes, thereby, suggesting that [EMIM][BF_4_] can be used as a suitable transducer for sensing of our target analytes. The CV peaks also point out the faradaic nature of the species which enables the diffusion from electrolyte to electrode surface. To get a better understanding of the role of diffusion current at the electrode electrolyte interface, we varied the scan rate from 25 mV/S to 250 mV/S for sensing both the analytes. The result is depicted in Figure 3.

An increasing trend in peak current as well as area under the curve was obtained suggesting that the presence of RTIL enables the charge diffusion process at the electrode electrolyte micro-environment, ultimately improving signal transduction. The peak current vs. scan rate was plotted with R^2^ for both the analytes 2,6 DNT and ENB and has been depicted in Figure S2a,b, respectively in the Supplementary Materials. The linear response supports the electrochemical microenvironment to be diffusion limited. Furthermore, to obtain the calibrated dose response, we have performed square wave voltammetry.

### 3.3. Square Wave Voltammetry (SQWV) Comparison and Calibration

**Figure 4:**
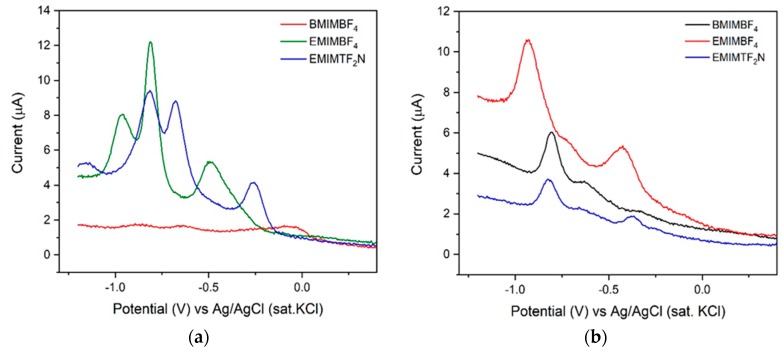
Square wave voltammetry has been performed using RTIL-modified GCE in two different analytes with a frequency of 25 Hz, amplitude of 25 mV and step size of 5 mV. (**a**) SQWV of different RTIL@GCE for (**a**) 2,6 DNT (**b**) ENB, showing higher peak current for [EMIM][BF_4_].

Square wave voltammetry (SQWV) is a powerful tool that can provide fingerprint redox properties of the compounds that are generally unable to show proper voltammetric responses. As we found different oxidation and reduction peaks for the two analytes in CV, we performed SQWV to build a calibration with a better resolution. The SQWV was performed using the RTIL-coated GCE. Different concentrations of two analytes: 2,6 DNT and ENB were prepared using 0.1 M KCl as supporting electrolyte. Analysis was performed from +0.4 V to −1.2 V with an amplitude of 10 mV, frequency of 25 Hz and step size of 5 mV. SQWV was undertaken to evaluate the performance of three different RTILs for sensing 100 ppm target analyte concentrations. The results are depicted in Figure 4.

**Figure 5:**
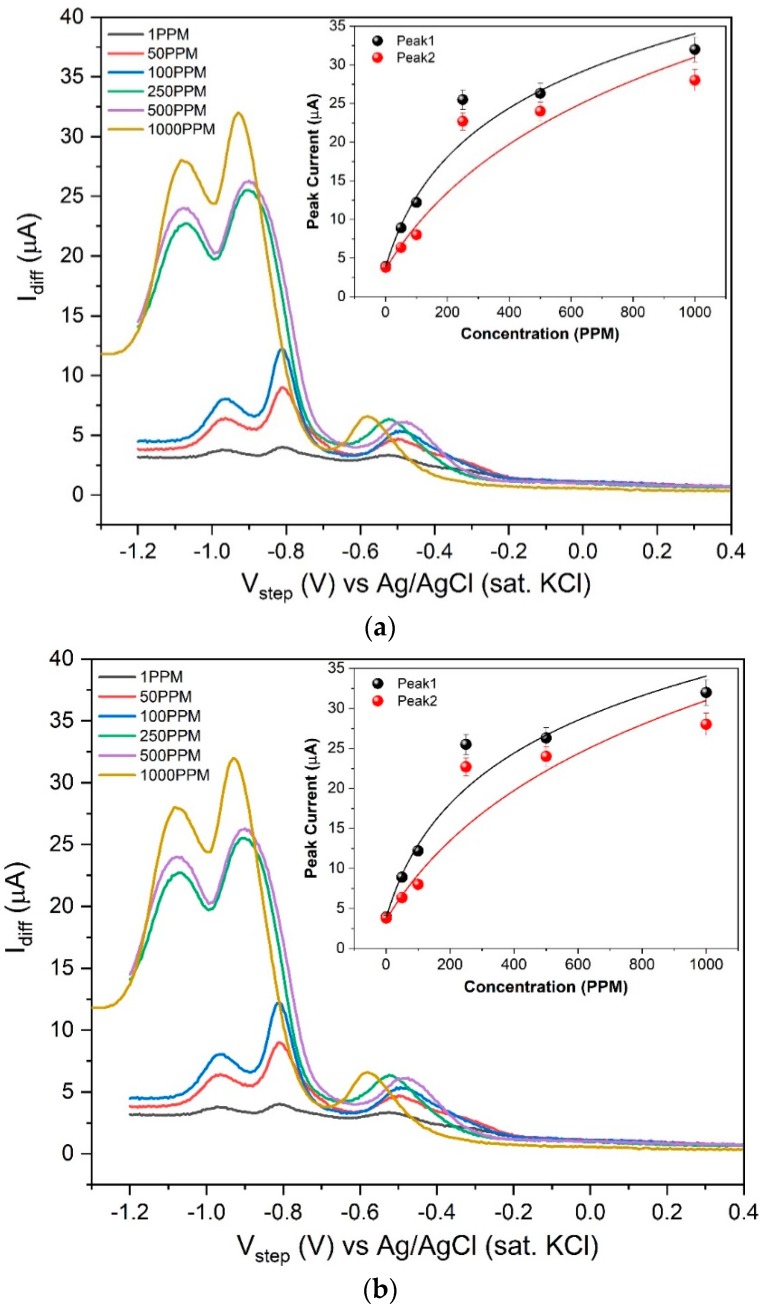
Square wave voltammetry performed using [EMIM][BF_4_]-modified GCE in two different analytes with varying concentration. SQWV parameter: frequency 25 Hz, amplitude of 25 mV and step size of 5 mV. Calibrated dose response of (**a**) 2,6 DNT showing two distinct peaks which varies with concentration (inset Figure 2a); (**b**) ENB showing one distinct peak which varies with concentrations. (inset Figure 2b).

Among the three RTILs, [BMIM][BF_4_] does not show any significant response for 2,6 DNT, whereas [EMIM][BF_4_] shows good current response for both the analytes. The peak current for nitro reduction using [EMIM][BF_4_]-coated GCE can be seen at −0.81 V and −0.96 V for 2,6 DNT and −0.94 V for ENB. The peaks −0.81 V and −0.94 V correspond to reduction of –NO_2_ to corresponding –NHOH [33]. Guo et al. [47] and Chen et al. [48] used differential pulse voltammetry to obtain –NO_2_ reduction and obtained a good dose-dependent response for the electrochemical detection of nitroaromatics. These results support our experimental results obtained for the reduction of nitroaromatics. A similar peak is present in ENB at −0.94 V which strengthens our claim of –NO_2_ reduction. The small peak near −0.4 V corresponds to the RTIL itself which can be seen in Figure S3 in the Supplementary Materials, where we performed the same experiment without any analyte. We also performed SQWV with only nafion, bare GCE and [EMIM][BF_4_]-nafion modified GCE for 100 ppm of 2,6 DNT and ENB and the result is depicted in Figure S4a,b, respectively in the Supplementary Materials. The result shows the efficacy of RTIL as signal mediator compared to electrode without an RTIL. The high ionic conductivity of the RTIL plays a crucial role in conducting the charge in the micro-environment which makes the system electrochemically responsive. The transducing effects of [EMIM][BF_4_] led us to build the calibrated dose response by varying the concentration from 1 ppm to 1000 ppm for both the analytes. The sensor response is plotted as current difference (forward-backward) with respect to the step potential. Two visible peaks corresponding to the reduction of –NO_2_ can be seen at −0.81 V and −0.96 V in Figure 5a for 2,6 DNT. We calculated the peak currents at these potentials corresponding to increasing concentrations. We achieved a good correlation of current vs. concentration, depicted in set of Figure 5a. The limit of detection is defined as the minimum amount of the target analyte that can be detected above the baseline. The limit of detection for the analyte is found to be 1 ppm. Moreover, the slope of the graph in Figure 5a for 2,6 DNT, for peak 1 37.38 nA/ppm and peak 2 is 31.49 nA/ppm. 

We found a similar calibrated dose response using [EMIM][BF_4_]-coated GCE for ENB and the result is depicted in Figure 5b. The peak current also shows good correlation with the concentration and the result is depicted in Figure 5b. Limit of detection is found to be 1 ppm and the slope of the graph in Figure 5b is 17.28 nA/ppm. 

The data reported were compiled with n = 3 and plotted with 5% error bar (95% confidence interval). The result obtained shows that [EMIM][BF_4_] acts as a suitable candidate for the sensing of nitroaromatic compounds with high sensitivity and redundancy. 

### 3.4. Open Circuit Potential

Open circuit potential (OCP) is an important characteristic to obtain the stability of the sensor. It can be defined as the summation of the half-cell reaction potentials for a specific electrode–electrolyte interface. The potentiostat measures the working electrode with respect to the reference electrode over time. A stable electrochemical sensor should have an OCP in the lower millivolt range when measured over time [49]. We performed OCP measurement for the three electrodes setup using 0.1 M KCl as the electrolyte. The working electrode is used as the [EMIM][BF_4_]-nafion network, coated over the GCE. The results are depicted in Figure S5 in the Supplementary Materials. An OCP of 250 mV was obtained over 200 s indicating the electrochemical stability of the sensor.

### 3.5. Portable Amperometric Interdigitated Electrode (IDE) Sensor for Detection of Nitroaromatic Compounds

**Figure 6:**
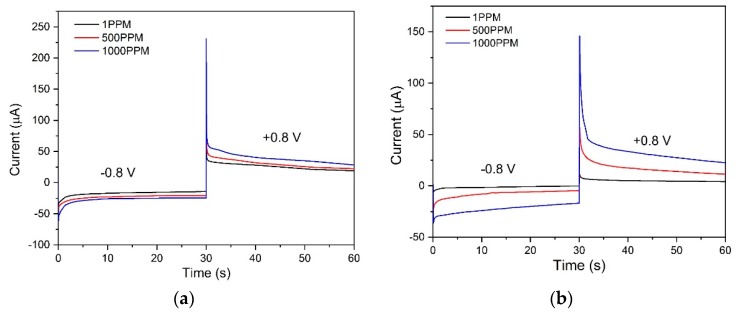
Double potential chronoamperometry scan was performed at −0.8 V for 30 s and +0.8 V for 30 s on the [EMIM][BF_4_]-nafion modified interdigitated electrode (IDE) for the two target analytes. Calibrated dose response chronoamperograms of (**a**) 2,6 DNT and (**b**) ENB which varies with concentration.

The standard three electrode setup showed promising data for the use of a specific RTIL as an electrochemical transducer and its application in the detection of nitroaromatics. This can easily be developed into a hand-held device for on-site detection. The IDE is a well-known miniaturized platform for its use in real-field applications and for its effectiveness in measuring diffusion as its interdigitated architecture results in a high surface-to-volume ratio. Our group introduced such a platform few years ago for real field detection of CO_2_ [50]. The selected IDE design uses a microliter volume of the sample, is easy to fabricate, robust in sensing, and allows for incorporation into a hand-held device. To obtain diffusion current, a double potential chronoamperometry scan was performed at −0.8 V for 30 s and +0.8 V for 30 s. Transient cathodic diffusion current was recorded at 5 s. A thin layer of RTIL-nafion network allows for easy diffusion of the reduced species towards the sensor surface and reduces interference thereby working as a good transducer for sensing [49]. Chronoamperometry is used as the electrochemical transduction technique to allow for the measurement of reduction current at the electrode–electrolyte interface by applying a step DC potential. The experiments were carried out for three concentrations, 1, 500, and 1000 ppm for n = 3 replicates. The chronoamperograms were recorded and are depicted in Figure 6. Three doses were chosen so that the sensor performance could be evaluated at low-, medium-, and high-dose concentrations of the target analytes. The results obtained show increasing cathodic and anodic current with increasing concentration due to a higher number of species being diffused at the electrode-electrolyte interface. Steady state current values at 5 s were recorded and the calibrated dose-response plots have been shown in Figure S6a,b in the Supplementary Materials for 2,6 DNT and ENB, respectively.

The GCE vs. IDE current magnitude at 5 s is reported in Figure S7 in the Supplementary Materials. The diffusion current in the IDE also supports our hypothesis that the species [EMIM][BF_4_] performs better in a field-deployable IDE platform.

## 4. Conclusions

In this study, we successfully performed the detection of nitroaromatic compounds using a GCE modified with a specific RTIL-nafion network. The work highlights the selection of a particular RTIL, that is, [EMIM][BF_4_], for the detection of nitroaromatic compounds as it amplifies the signal and the [EMIM][BF_4_]-nafion network acts as a transducer. The nitroaromatic compounds such as 2,6-DNT have two –NO_2_ groups which can be reduced easily into hydroxylamine and the reduction peaks for both groups can be seen, whereas for ENB, which contains only one nitro group, the reduction peak appears only once. The phenomenon of electrochemical reduction of nitroaromatic compounds is a complex procedure that is dictated by variety of factors such as the number of nitro groups present, the position at which they are present, and the effect of other substituents on the benzene ring system. The results obtained helped in identifying the type of anion and cation suitable for the detection of nitroaromatics and can be employed using an IDE that can be incorporated easily into a hand-held device. This platform can help in the field monitoring of nitroaromatic compounds with a concentration as low as 1 ppm. We leveraged the electrochemical properties of [EMIM][BF_4_] for the signal amplification and developed an electrochemical sensor that can serve as a robust and sensitive platform for the identification of nitroaromatic compounds. 

## Figures and Tables

**Table 1:** Conductivity and electrochemical stability of the room-temperature ionic liquids (RTILs) under investigation.

RTIL	Conductivity	Electrochemical Stability
[EMIM][BF_4_]	12 mS/cm	4.3 V
[BMIM][BF_4_]	10 mS/cm	4.0 V
[EMIM][TF_2_N]	11 mS/cm	3.8 V

**Table 2:** Cathodic and anodic peak potential and peak current for 2,6 DNT obtained from Figure 2a.

Compound	$E_{p}^{a}$ (V)	$E_{p}^{c}$ (V)	$I_{p}^{a}$ (µA)	$I_{p}^{c}$ (µA)
[BMIM][BF_4_]	−0.036	−0.504, −0.816, −0.995	3.39	−5.95, −16.39, −18.41
[EMIM][BF_4_]	−0.137	−0.221, −0.697, −0.837	5.14	−2.91, −14.28, −19.04
[EMIM][TF_2_N]	−0.205	−0.291, −0.717, −0.870	4.56	−1.61, −12.82, −17.60

**Table 3:** Cathodic and anodic peak potential and peak current for ENB obtained from Figure 2b.

Compound	$E_{p}^{a}$ (V)	$E_{p}^{c}$ (V)	$I_{p}^{a}$ (µA)	$I_{p}^{c}$ (µA)
[BMIM][BF_4_]	−0.120	–0.634, −0.804	2.26	−6.41, −7.63
[EMIM][BF_4_]	−0.234	–0.482, −0.929	2.26	−7.65, −14.06
[EMIM][TF_2_N]	−0.164	–0.362, −0.819	1.48	–1.97, −3.716

## Supplementary Materials

The following are available online at https://www.mdpi.com/1424-8220/20/4/1124/s1: Figure S1: Cyclic voltammetry (CV) output of room-temperature ionic liquid (RTIL)-modified glassy carbon electrode (GCE) without analyte, Figure S2: (a) The peak current vs. scan rate with R2 for 2,6-dinitrotoluene (2,6 DNT); (b) The peak current vs. scan rate with R2 for ethylnitrobenzene (ENB), Figure S3: Square wave voltammetry (SQWV) was performed using different RTILs in the absence of the target analyte to obtain the baseline peak current. SQWV parameter: frequency 25 Hz, amplitude of 25 mV and step size of 5 mV, Figure S4: (a) SQWV output of RTIL ([EMIM][BF4])-nafion modified GCE, bare electrode and nafion-GCE for detection of 100 ppm 2,6 DNT; (b) SQWV output of RTIL ([EMIM][BF4])-nafion modified GCE, bare electrode and nafion-GCE for detection of 100 ppm ENB, Figure S5: Open circuit potential measurement to show the stability of the fabricated sensor. The sensor is stable for over 200 s with the OCP in the lower millivolt regime, Figure S6: (a) Calibration dose response plotted for 2,6 DNT concentrations of 1, 500 and 1000 ppm in terms of steady state current at 5 s; (b) Calibration dose response plotted for ENB concentrations of 1, 500 and 1000 ppm in terms of steady state current at 5 s, Figure S7: Comparison of the current obtained at 5 s for interdigitated electrode (IDE) vs. GCE for both the target analytes.
