# Supplementary material for: Characterization of Room-Temperature Ionic Liquids to Study the Electrochemical Activity of Nitro Compounds
Source: Sensors (Basel). 2020 Feb 19;20(4):1124. doi: 10.3390/s20041124 (PMC7070553; doi:10.3390/s20041124)
Supplement: Supplementary file 1 [file sensors-20-01124-s001.pdf]

sensors

MDPI

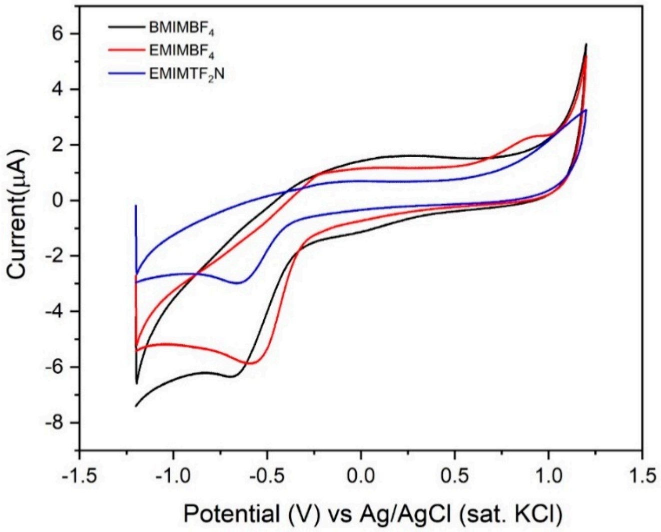

Figure S1. Cyclic voltammetry (CV) output of room-temperature ionic liquid (RTIL)-modified glassy carbon electrode (GCE) without analyte.

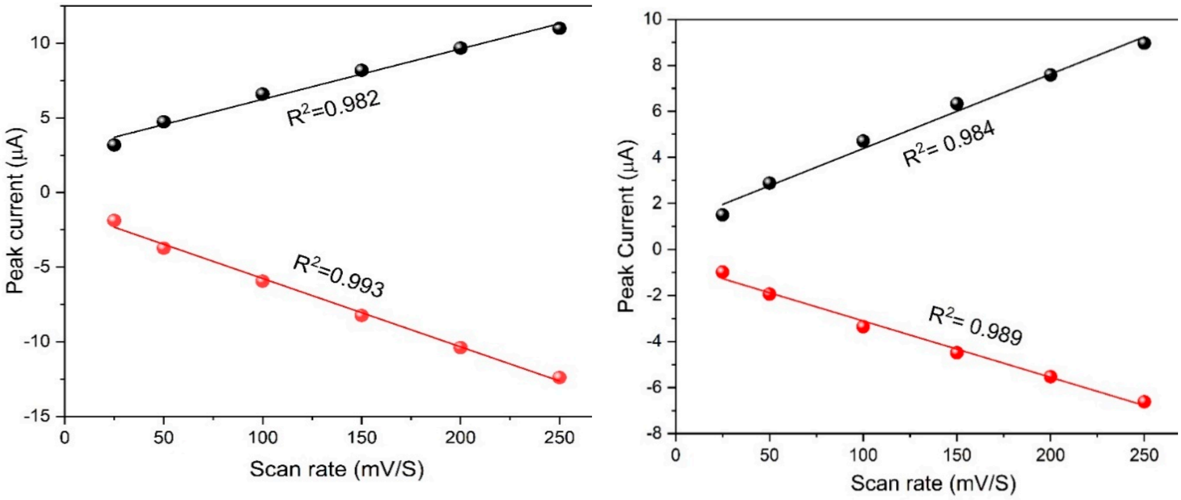

(**a**) (**b**)

Figure S2. (**a**) The peak current vs. scan rate with R^2 for 2,6-dinitrotoluene (2,6 DNT); (**b**) The peak current vs. scan rate with R^2 for ethylnitrobenzene (ENB).

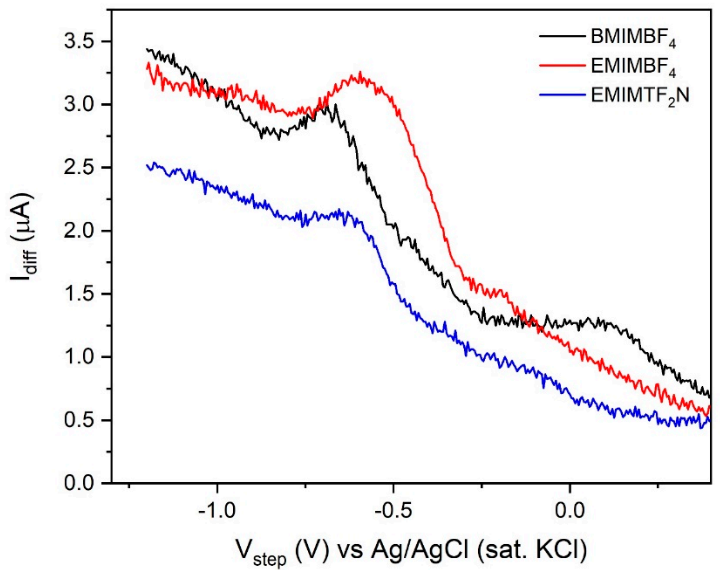

Figure S3. Square wave voltammetry (SQWV) was performed using different RTILs in the absence of the target analyte to obtain the baseline peak current. SQWV parameter: frequency 25 Hz, amplitude of 25 mV and step size of 5 mV.

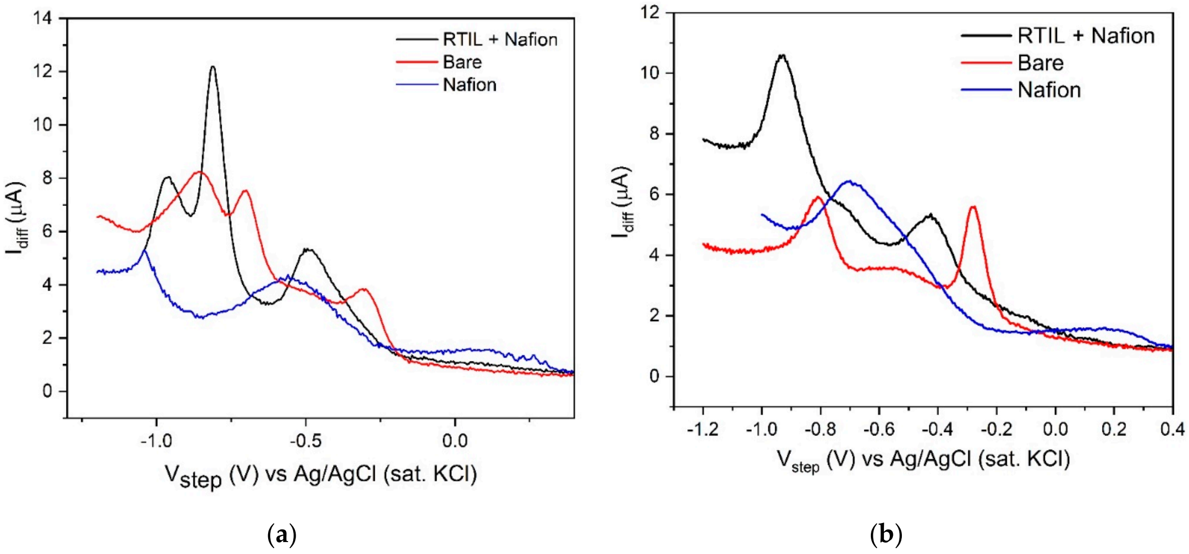

Figure S4. (**a**) SQWV output of RTIL ([EMIM][BF_4])-nafion modified GCE, bare electrode and nafion-GCE for detection of 100 ppm 2,6 DNT; (**b**) SQWV output of RTIL ([EMIM][BF_4])-nafion modified GCE, bare electrode and nafion-GCE for detection of 100 ppm ENB.

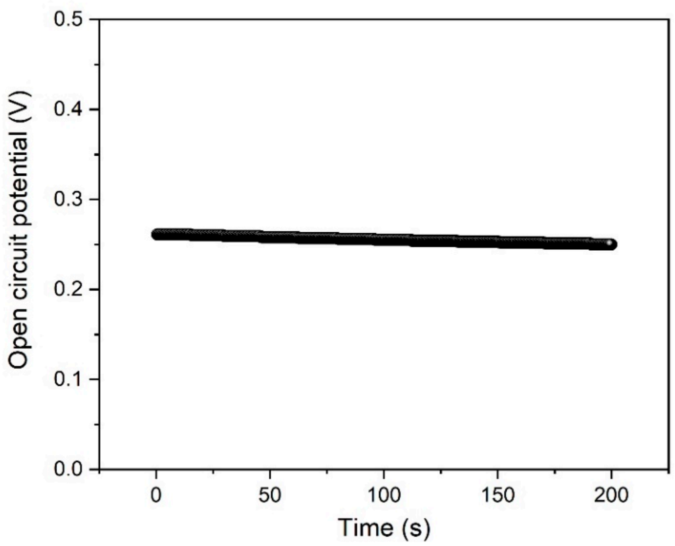

Figure S5. Open circuit potential measurement to show the stability of the fabricated sensor. The sensor is stable for over 200 s with the OCP in the lower millivolt regime.

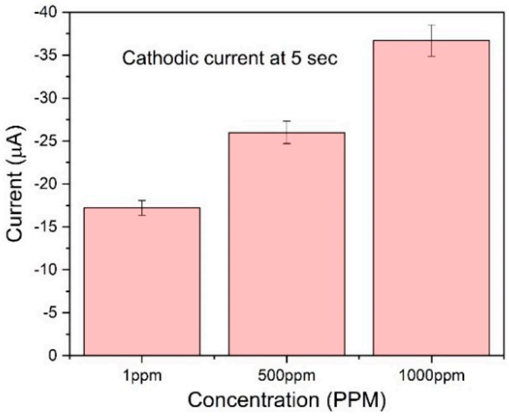

(**a**)

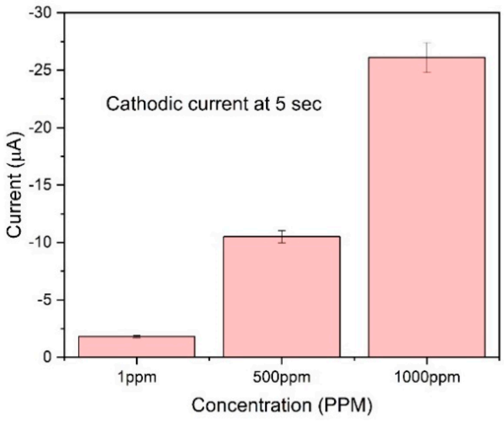

(**b**)

Figure S6. (**a**) Calibration dose response plotted for 2,6 DNT concentrations of 1, 500 and 1000 ppm in terms of steady state current at 5 sec; (**b**) Calibration dose response plotted for ENB concentrations of 1, 500 and 1000 ppm in terms of steady state current at 5 sec.

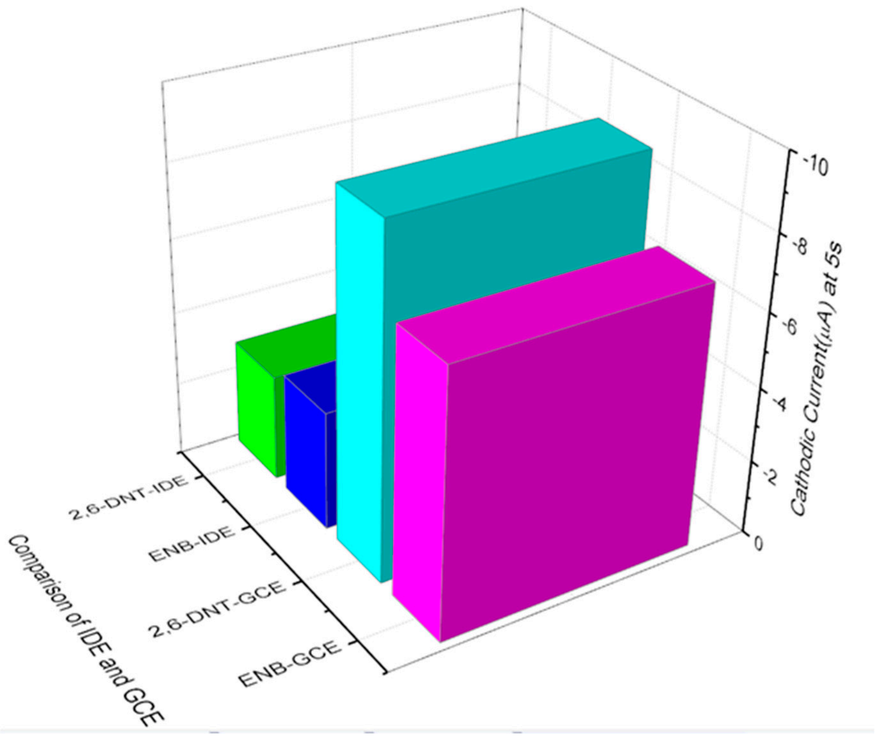

Figure S7. Comparison of the current obtained at 5 s for interdigitated electrode (IDE) vs. GCE for both the target analytes.

© 2020 by the authors. Licensee MDPI, Basel, Switzerland. This article is an open access article distributed under the terms and conditions of the Creative Commons Attribution (CC BY) license (http://creativecommons.org/licenses/by/4.0/).
